# Biomechanical and histomorphometric evaluation of skin integration on titanium and PEEK implants with different surface treatments

**DOI:** 10.1007/s10856-022-06687-y

**Published:** 2022-09-30

**Authors:** Per Kjellin, Karin Danielsson, Joakim Håkansson, Karin Agrenius, Therese Andersson, Patrik Stenlund

**Affiliations:** 1 Promimic AB, AstraZeneca BioventureHub, SE 43183 Mölndal, Sweden; 2 Department of Methodology, Textile and Medical Technology, RISE Research Institutes of Sweden, SE 50115 Borås, Sweden; 3 Department of Laboratory Medicine, Institute of Biomedicine, Gothenburg University, SE 40530 Göteborg, Sweden

## Abstract

Percutaneous implants are frequently affected by bacterial growth at the skin-implant interface. Integration between implant and surrounding skin is important to prevent bacteria from spreading to the underlying tissue. The standard method to evaluate skin-implant integration is by histomorphometry on samples which have been placed in tissue grown in vivo or ex vivo. In this study, a biomechanical method was developed and evaluated. The integration of implants into porcine skin was studied in an ex vivo model, where pig skin samples were cultivated in a nutrient solution. Cylindrical shaped implants, consisting of polyether ether ketone (PEEK) and titanium (Ti) with different surface treatments, were implanted in the skin tissue and the skin was grown in nutrient solution for 2 weeks. The implants were then extracted from the implantation site and the mechanical force during extraction was measured as a quantitative assessment of skin-implant integration. Implants from each group were also processed for histomorphometry and the degree of epidermal downgrowth (ED) and tissue to implant contact (TIC) was measured. A higher mean pullout force was observed for the PEEK implants compared to the Ti implants. Applying nanosized hydroxyapatite (HA) on Ti and PEEK increased the pullout force compared to uncoated controls, 24% for machined and 70% for blasted Ti, and 51% for machined PEEK. Treatment of Ti and PEEK with nanosized zirconium phosphate (ZrP) did not increase the pullout force. The histomorphometry analysis showed correlation between ED and pullout force, where the pullout force was inversely proportional to ED. For TIC, no significant differences were observed between the groups of same material (i.e. Ti, Ti+HA, Ti+ZrP, and PEEK, PEEK + HA, PEEK + ZrP), but it was significantly higher for PEEK compared to Ti. Scanning electron microscopy analysis was done on samples before and after the pullout tests, showing that the ZrP coating was unaffected by the 2 week ex vivo implantation and pullout procedure, no dissolution or detachment of the coating was observed. For the HA coating, a loss of coating was seen on approximately 5% of the total surface area of the implant.

**Figure:**
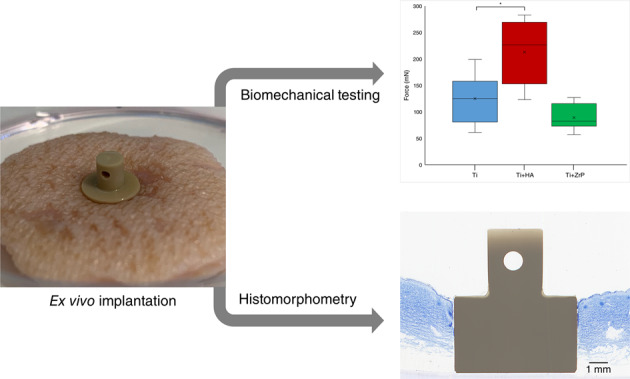
Graphical abstract

Graphical abstract

## Introduction

Percutaneous devices are used to treat a wide range of medical conditions. Examples of percutaneous devices include metallic implants such as external fixation pins [1], bone anchored hearing aids [2] and bone anchored prostheses [3], and also polymeric devices such as peritoneal catheters [4] and gastrostomy tubes [5]. One complication that may occur with percutaneous implants is bacterial growth at the skin-implant interface. Since a percutaneous device penetrates the skin, bacteria can bypass the protective skin barrier by growing along the implant surface and enter the underlying tissue. This may lead to serious infections and, if the implant is anchored in bone, conditions such as osteomyelitis [6, 7]. For dental abutments, which are positioned between the bone-anchored implant screw and the dental crown, bacterial growth onto the abutment and subsequent spreading of the bacteria to the jawbone may cause implant loosening, so called periimplantitis [8].

There are different strategies to prevent bacterial downgrowth on percutaneous implants. Preventing bacterial adhesion is one approach, where stainless steel fixation pins for example are polished to a very high degree of smoothness. The idea behind this treatment is that a smooth surface leaves less surface area for bacteria to grow on. Another strategy is to apply an antibacterial coating onto the implant [9]. Yet another approach is to enable the skin to integrate with the implant surface, thereby forming a seal which prevents bacteria from penetrating the skin barrier through the skin-implant interface. This can be achieved by changing the implant surface topography with blasting or acid etching, or by applying some type of coating. Hydroxyapatite (HA) has shown a beneficial effect on soft tissue integration, for example when used as a coating on bone anchored hearing aids [10] or on external fixation pins [11–13], or as a solid sintered material [14, 15]. Titanium dioxide has also shown promising results [16].

For bone tissue integration, biomechanical testing of the anchoring strength of the implant is a common evaluation tool. This can be done by various methods, such as by measuring the removal torque of the implants [17] or by pullout or pushout tests [18, 19]. Biomechanical testing on soft tissue is, on the other hand, not as easy to perform. Factors that complicate the measurement are low adhesive forces during the measurement and difficulties of keeping the implant stabilized during the healing process, which tend to cause a large standard deviation for biomechanical testing on soft tissue [20]. The most common method to study and evaluate soft tissue response on implants is therefore by performing histomorphometry, using dyes such as hematoxylin and eosin to selectively stain the tissue.

When an implant is not accepted by the surrounding skin, the skin starts to grow downwards and along the implant surface, thereby sealing off the skin from the implant surface. This process, called epidermal downgrowth (ED) is normally regarded as the key factor when determining implant / skin compatibility; a low ED indicates that the implant is a suitable material for soft tissue integration and vice versa [21–23]. Another parameter, less frequently measured, is the tissue to implant contact (TIC), which is the length (or percentage of the tissue height) of the tissue that is in close contact to the implant [13, 24].

Histomorphometry has the primary advantage of enabling study of the implant–tissue interface in high magnification, which gives valuable knowledge of the tissue appearance. It is also possible to gain information on the type of tissue which has formed during implantation and the status of this tissue. However, histomorphometry is an expensive technique and the number of processed slides is therefore kept low in a typical study. Since the thickness of a slide is usually 25–50 µm, each slide constitutes a very small amount of the total surface area of the implant; for an implant which is 5 mm in diameter, a microscopy slide with a thickness of 25 µm will consequently display only 0.5% of the total surface, and for each implant sample it is not uncommon to do only 1 or 2 slides. Biomechanical testing, such as removal torque, pull-out or push-out, does not give any information on the appearance of the tissue at the implant-tissue interface, but it has the advantage that results are obtained rapidly, corresponds to the entire interfacial area, and gives information of high clinical relevance; how strong the implant is attached to the surrounding tissue. Ideally, a combination of biomechanical testing and histomorphometry should be used to give information on the tissue-implant interaction.

Evaluating the implant interaction with skin by using an ex vivo model has the benefit of being more controlled since the position of the implant and the conditions during implantation can be closely monitored. Another advantage is that it is efficient; large amounts of skin can be harvested from one animal, meaning that more tests per animal can be performed. From an ethical point of view it is also highly desirable to reduce the number of test animals as much as possible [25]. The disadvantages with the ex vivo model is that the skin is grown in a petri dish, and has no operating blood vessels which means that it is highly likely that the skin growth will be slower compared to in vivo conditions [26]. There is also a limit on how long explanted skin can be kept alive and still resemble in vivo skin properties. Cultivating skin ex vivo more than 2–3 weeks is usually not meaningful [27, 28], which means that long term studies (i.e. several months) are not possible to conduct with current state of the art ex vivo models. In this study, skin samples obtained from pigs were used for implantation since pig skin resembles human skin and is frequently used for soft tissue studies [29, 30].

The main purpose of this study was to investigate if it was possible to measure the degree of skin-implant integration by using biomechanical (pullout) testing in an ex vivo tissue model. The results were compared with histomorphometry, measuring both ED and TIC. Titanium (Ti), which is by far the most common implant material and is frequently used for soft tissue applications, was one of the tested substrates. Polyether ether ketone (PEEK), which is a polymeric material frequently used in orthopedics, especially in spinal fusion cages, was also included. PEEK is well accepted by bone tissue but is generally considered as having a relatively poor effect on bone tissue growth in comparison with Ti [31–33]. The behavior in soft tissue for PEEK is not extensively studied, but it could be a promising material for soft tissue integrating devices due to its excellent chemical resistance, ease of manufacturing and high mechanical strength [34].

Two types of coatings were applied on top of the Ti and PEEK substrates: HA and zirconium phosphate (ZrP). HA was included in the study since, as mentioned previously, it has a documented effect on soft tissue attachment. In this study, a nanosized HA coating was used, which does not change the roughness on the micrometer scale [35, 36]. ZrP is another material that can be applied as a thin coating, and similar to HA it stimulates bone tissue growth [37]. However, the behavior of ZrP in soft tissue has, to our knowledge, not been studied previously.

## Materials and methods

### Preparation of implants

**Fig. 1:**
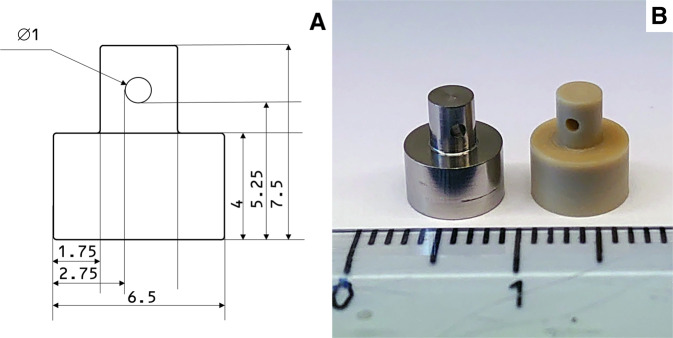
**A** Drawing of the implant. Measurements in mm. **B** Photo of a Ti (left) and PEEK (right) implant

A drawing of the implant is shown in Fig. 1A. The implant consisted of a cylindrical section with a diameter of 6.5 mm, a height of 4 mm and a smaller cylindrical section with a diameter of 3 mm and a height of 3.5 mm. A hole of 1 mm in diameter was drilled through the middle of the small cylinder to make it possible to attach the implant to the mechanical testing device. A photo of a Ti and a PEEK implant (both machined and non-blasted) is shown in Fig. 1B. The Ti implants were produced from Ti grade 4, and the PEEK implants from PEEK-Optima (Invibio Ltd). For the blasted Ti implants in the first test, the Ti implants were blasted with Al_2_O_3_ with a grit size of 200–250 µm, using a pressure of 0.6 MPa, a blasting time of 30 s and a distance between nozzle and implant of 10 cm. The HA coating of the implants was applied according to [36]. The ZrP coating was applied as described in [37].

### Surface characterization

The surface topography was analyzed with white light coherence scanning interferometry using a MicroXAM from ADE Phase Shift Technology, USA, with a magnification of 50X and a measurement area of 160 × 125 µm. Two implants from each group and 3 randomly selected spots on the test area (i.e., the area designed to be in contact with the skin) on each implant were analyzed. Scanning electron microscopy (SEM) was performed with a Zeiss Gemini 300 dual beam instrument, using an acceleration voltage between 1.5 and 2.5 kV and a secondary electron or an InLens detector, depending on the samples. Prior to the SEM analysis, the PEEK samples were sputtered with gold in a JEOL JFC-100E sputter, using a current of 10 mA for 100 s.

### Collection of skin samples and in vitro cultivation

**Fig. 2:**
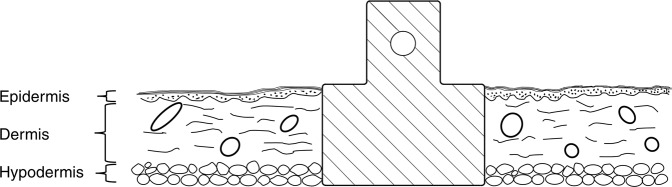
Illustration of the implant placed in skin

**Fig. 3:**
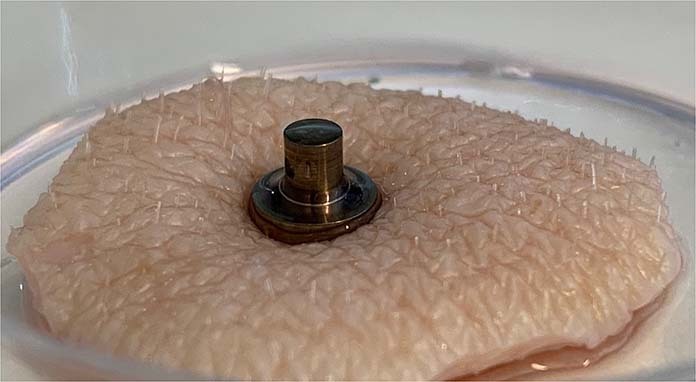
Photograph of an implant inserted in skin

The pigs used in the tests was a mixed breed of Yorkshire, Hampshire and Swedish Pigham. The pigs were used in another research study and the skin was explanted after euthanization, so no additional ethical permission was required for this study. Directly after euthanization, the pig was shaved with a clipper and washed with Hibiscrub (Mölnlycke Health Care, Sweden), and ethanol (CCS Healthcare). The skin was taken from the back of the pig and transported to the lab in a plastic box containing sterile saline (0.9% NaCl (Fresenius Kabi)). The subcutaneous fat was removed using a scalpel and the skin was cleaned with chlorohexidine (Fresenius Kabi) and then once more with alcohol wipes and shaved using a scalpel. The skin tissue was thereafter immersed in DPBS containing 1% PEST (Penicillin and Streptomycin), 1 µg/l Amphotericin B, 20 µg/l Gentamicin and 20 µg/l Piperacillin (Gibco™) for 1 h. Circular tissue samples, approximately Ø 3.5 cm, were then cut out from the skin tissue by a scalpel. A biopsy punch (Ø 6 mm) was used to create a circular full thickness wound in each tissue sample. The tissue samples were placed in petri dishes (one tissue/dish). Figure 2 shows an illustration of how the implant was positioned in the skin; Fig. 3 shows a photograph of a Ti implant inserted in the circular skin piece. The low density of PEEK compared to Ti resulted in a large difference in weight between Ti and PEEK implants; 0.71 g compared to 0.22 g, respectively. This difference caused the PEEK implant-skin samples to float in the medium, whereas the Ti implant and skin rested firmly on the bottom of the petri dish. As this difference could potentially affect the experimental results, the PEEK implants were pushed down by placing a stainless steel nut (weight 0.6 g) on top of the PEEK implants. This piece was not in contact with the skin-implant interface. To each petri dish, 10 ml DMEM/F-12 + GlutaMAX^TM^ (Gibco™) supplemented with 10% FBS and 1% PEST (Penicillin and Streptomycin), 1 µg/l Amphotericin B, 20 µg/l Gentamicin and 20 µg/l Piperacillin was added, and the tissues were cultured at 37 °C and 5% CO_2_, for 14 days. The medium was changed every other or third day (day 2, 4, 7, 9, 11, and 13). For the test including histomorphometric evaluation, 2 samples in each group were selected for histology analysis and placed in neutral buffered formaldehyde (NBF). As controls, to monitor that the skin did not enter necrosis during cultivation, punch biopsies or scalpel incisions were made to separate skin pieces without any implants and cultivated in the same way as described above. Pictures were taken of the punch biopsy repeatedly during the cultivation and the incision was inspected at the end of the study to observe possible re-adhesion between the tissue surfaces.

### Histological processing

Samples for histology were taken from cultivation and placed in containers with neutral buffered formaldehyde (NBF). After a couple of hours, the NBF was replaced with new solution and the fixation was continued with periodical stirring for 48 h. The NBF was then replaced with water (Type 1), and the samples were placed in vials containing 70 wt % ethanol. The samples were subsequently dehydrated with new ethanol solutions with increasing concentration, 80, 95 and lastly 99.5%. They were reinfiltrated in diluted Technovit 7200 VLC followed by infiltration in pure Technovit 7200. The samples were embedded in pure Technovit 7200 VLC and hardened in a polymer hardening device (Histolux Kulzer, Tyskland). The polymer embedded tissue samples were split centrally with a water-cooled diamond laden sawblade followed by a renewed infiltration in Technovit 7200. The cut was subsequently hardened in daylight. Two central cuts with a thickness of 200–300 µm were obtained from each sample. These cuts were then polished down with wet sandpaper (grit size 1200) to approximately 30–40 µm thickness. Each cut was cleaned with 4% ExtranMA 01 solution followed by staining with Toluidine blue mixed with pyronin G and borax, according to [38]. After allowing the slides to dry, they were mounted with cover glass and Pertex (Histolab products AB, Gothenburg, Sweden).

### Biomechanical measurements

**Fig. 4:**
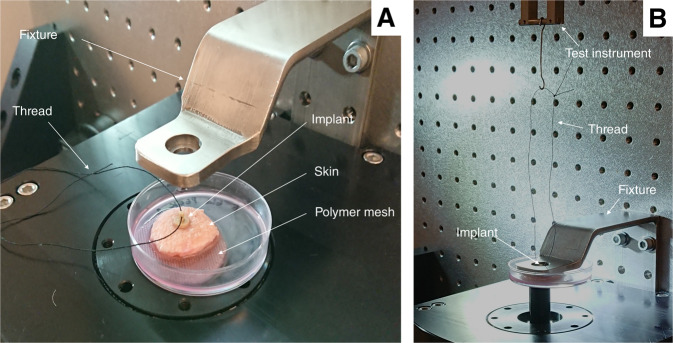
**A** Components of the pullout setup, with PEEK implant placed in skin and ready for testing, **B** Start of pullout test; implant is held in place by the fixture and the thread is connected to the test instrument

Table 1 shows a summary of the tests. Images of the test setup are shown in Fig. 4A, B. After two weeks of cultivation, the skin samples with inserted implants were carefully transferred from the nutrient solution to a clean petri dish. A polyethylene mesh with 0.3 mm pore size (Fisher Scientific) was placed at the bottom of the petri dish to decrease the risk of vacuum forming beneath the skin and implant, which could otherwise affect the measurement. A polyester thread (Gütermann M782) was attached to the implant in one end and to a Planar Biaxial TestBench Instrument (TA Instruments - ElectroForce System Group, Eden Prairie, MN, USA) in the other end. A fixture with a central hole, 8 mm in diameter, was used to hold the skin in place during the pullout testing. The implants were subjected to a preload of 5 mN and thereafter pulled upwards at a rate of 2.5 mm/min while simultaneously recording the force data using a 22 N (5 lbs) load cell at 20 Hz.

**Table 1:** Overview of ex vivo tests

Test	Substrates	Surfaces	Samples in each group	Evaluation
1	Blasted Ti	Ti, Ti+HA, Ti+ZrP	8 (pullout)	Interferometry, pullout, SEM
2	Machined Ti, machined PEEK	Ti, Ti+HA, Ti+ZrP, PEEK, PEEK + HA, PEEK + ZrP	8 (pullout), 2 (histo)	Interferometry, pullout, histomorphometry, SEM

### Histomorphometry

**Fig. 5:**
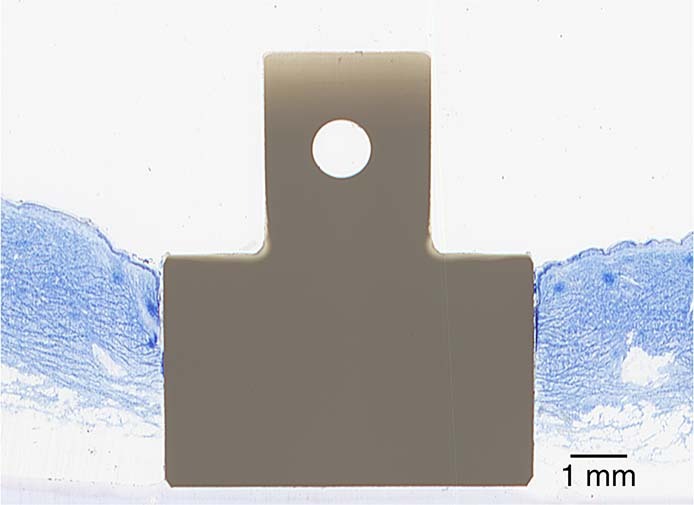
Histology slide, stained with toluidine blue, showing a PEEK implant (unmodified) placed in the skin

**Fig. 6:**
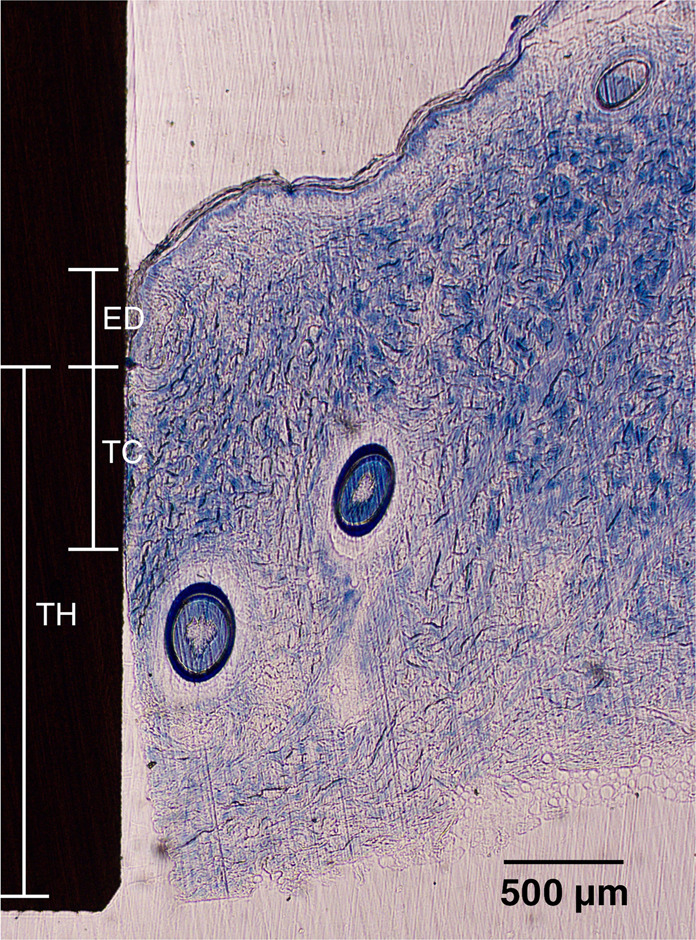
Histology slide, stained with toluidine blue, of an implant and surrounding skin. ED Epidermal downgrowth, TC Tissue contact length, TH Tissue height. TIC was calculated as TC/TH

**Fig. 7:**
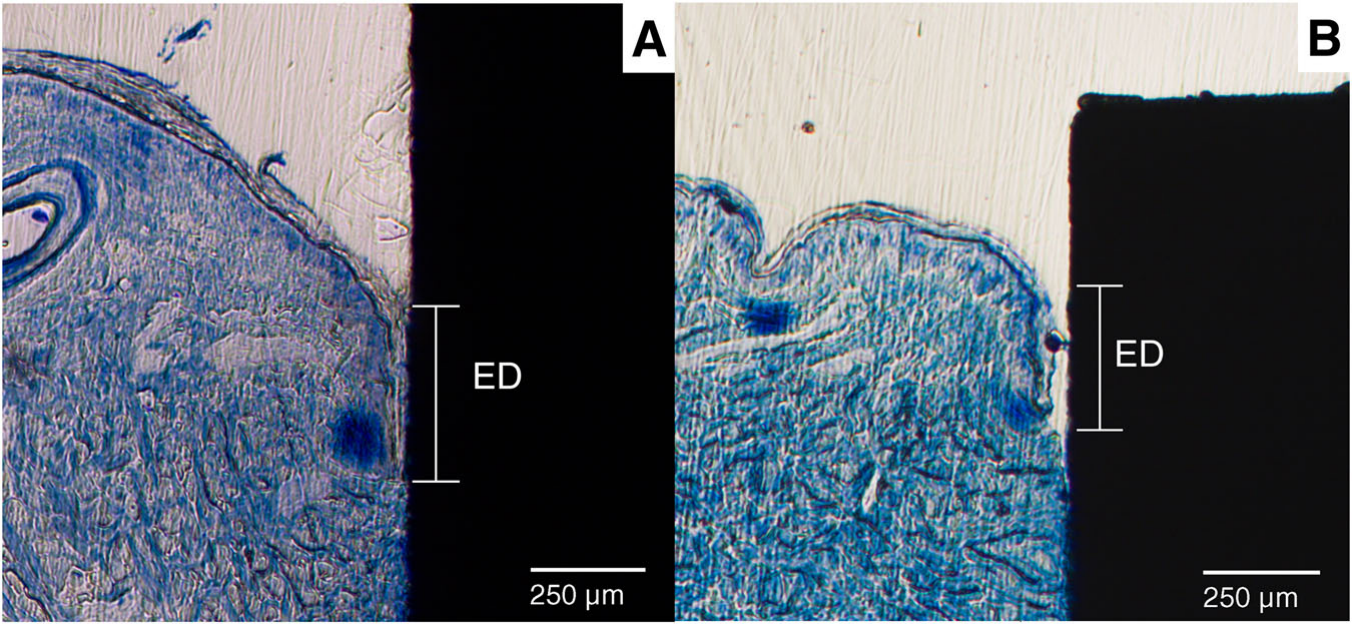
Histology slides showing different cases of Epidermal Downgrowth, **A** The downgrowth starts at the first contact point between skin and implant, **B** The start of the downgrowth is less defined, leading to a larger measurement error

A Zeiss Axioskop 40 was used to obtain images from the histological slides. The software ImageJ (available from the National Institutes of Health) was used for the image analysis. Figure 5 shows a low magnification histology slide of a PEEK implant placed in the skin. The slides were analyzed for ED and TIC. Figure 6 shows a histology slide (HA on PEEK) illustrating how the different integration parameters were defined. The term TC is used for tissue contact, referring to the length of the tissue in direct contact with the implant surface. TH refers to the tissue height below the ED. TIC was calculated as TC/TH. A complicating factor when measuring ED is the fact that the skin is not a planar surface. If the skin next to the implant would be perfectly flat and then grow downwards along the implant surface in a straight line, the measurement of ED would be straightforward, with a low level of experimental error. While it is relatively easy to determine where the downgrowth stops, determining the start of the downgrowth is less precise. Figure 7A shows a tissue sample of uncoated Ti with a well-defined epidermal downgrowth which occurred in a straight line and with a clear change in the skin curvature where the downgrowth starts. In contrast, Fig. 7B shows a tissue sample of HA coated Ti where the transition to epidermal downgrowth occurred over some distance, and the measurement error will hence be larger compared to in Fig. 7A. The TIC parameter is, compared to ED, more defined, as seen in Fig. 6.

### Examination of coated samples after implantation and extraction

The implant to be analyzed was placed in a beaker of 50 ml of DMSO (Fisher, ≥ 99.7%) and cleaned in an ultrasonic bath (Branson Sonorex) by ultrasonication for 60 min. Thereafter, the samples were washed extensively with 2-propanol (Fisher, ≥ 99.5%) to remove the DMSO and any loosely attached biological residues, before being analyzed with SEM.

### Statistical analysis

The biomechanical and histomorphometric results were statistically evaluated with the Mann-Whitney U test. *P*-values < 0.05 (two-tailed) were considered significant. For the biomechanical evaluation, 8 samples in each group were tested. For the histomorphometric analysis, 2 samples in each group were collected, and 2 slides were prepared for each sample, resulting in 4 slides per group. For each slide, both the left and right implant-skin area was analyzed.

## Results

### Surface characterization of samples

#### SEM

**Fig. 8:**
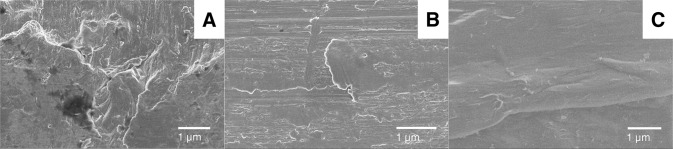
SEM images of the substrates: **A** Blasted Ti, **B** Machined Ti, **C** Machined PEEK

Figure 8 shows SEM images of the different substrates used in the study; (A) blasted Ti, (B) machined Ti, (C) machined PEEK. The blasted Ti surface displayed concave features such as scratches and pits, which are typical effects of the blasting treatment. The machined Ti surface was smoother, with visible striation patterns from the turning procedure. The PEEK surface had machining patterns with rounded edges, small polymeric fibers were also present on the surface.

**Fig. 9:**
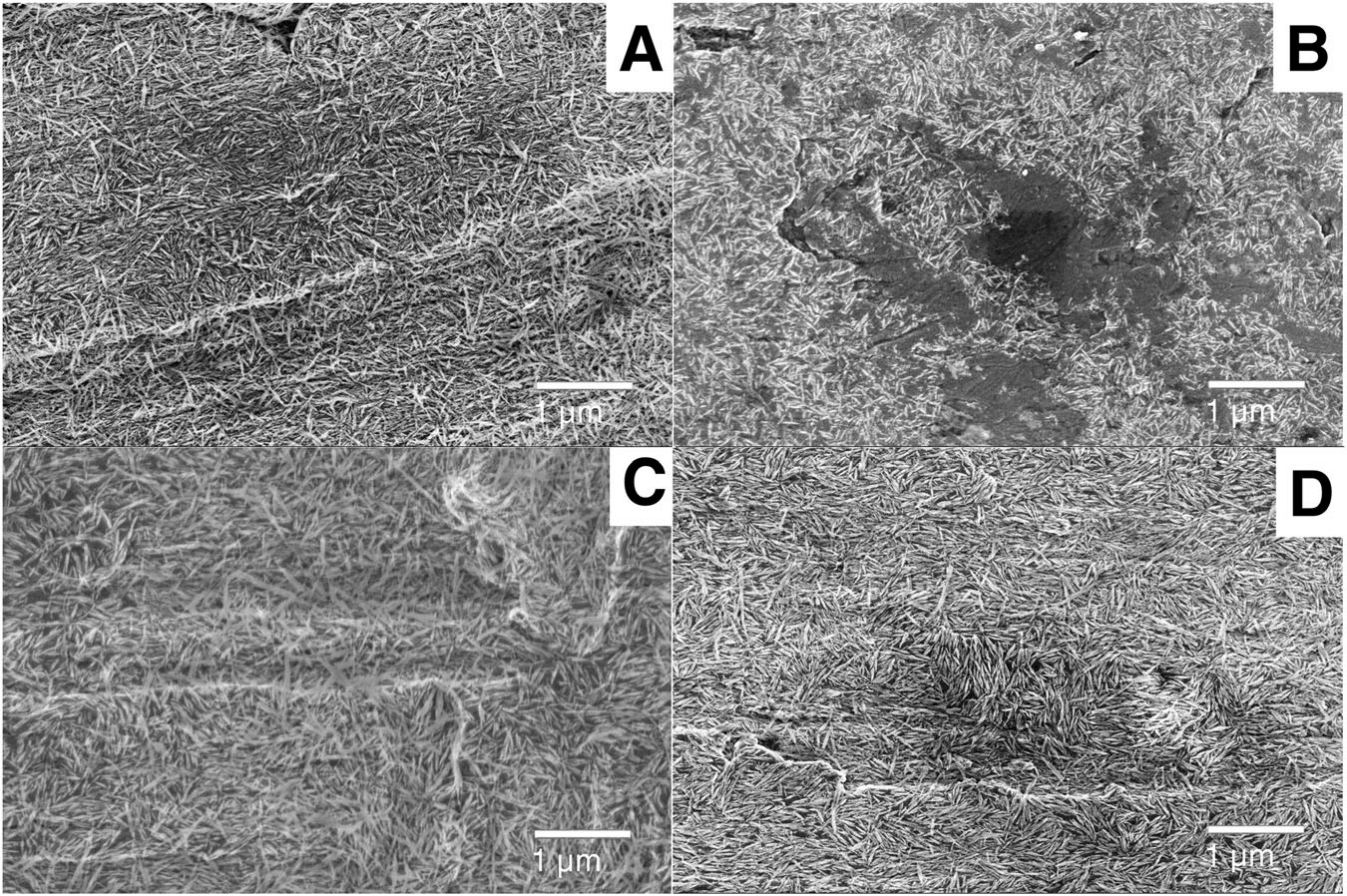
SEM images of coated Ti surfaces prior to and after insertion in the skin: **A** Blasted Ti+HA before insertion, **B** Blasted Ti+HA after insertion, **C** Machined Ti+HA before insertion, **D** Machined Ti+HA after insertion

**Fig. 10:**
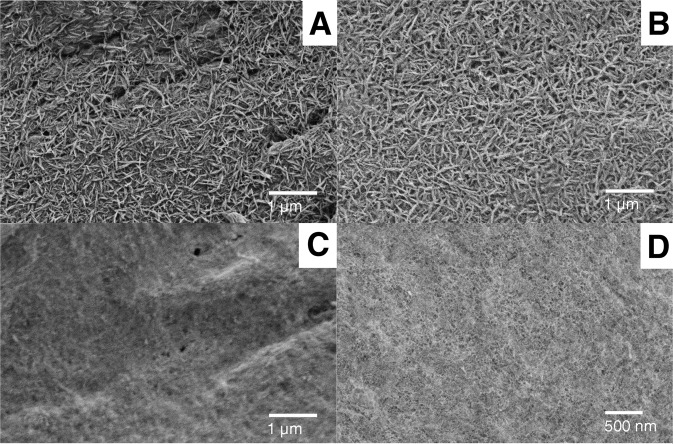
SEM images of coated PEEK surfaces, prior to and after insertion in the skin: **A** PEEK + HA before insertion, **B** PEEK + HA after insertion, **C** PEEK + ZrP before insertion, **D** PEEK + ZrP after insertion

The HA and ZrP coatings had the same appearances on the Ti and PEEK substrates; the HA coated samples appeared as a thin homogenous layer of needle shaped crystals, 5–20 nm wide and 100–200 nm long. The ZrP coating was visible as a porous layer on top of the substrate, with pores being 10–40 nm in diameter. Figure 9 shows a collection of SEM images of the HA coated Ti samples (ZrP coated Ti is not shown), before and after being inserted in the skin for 2 weeks and washed according to Section 2.7. 9A) blasted Ti+HA before insertion, B) blasted Ti+HA after insertion, C) machined Ti+HA before insertion, D) machined Ti+HA after insertion. Figure 10 shows a collection of SEM images of the coated PEEK samples, 10A) PEEK + HA before insertion, B) PEEK + HA after insertion, C) PEEK + ZrP before insertion, D) PEEK + ZrP after insertion. On the HA coated samples (Ti and PEEK), coating loss was observed in some areas, randomly distributed on the implant surface. Figure 9B shows one example of such an area, in which the HA crystals are gone, and the bare Ti substrate is visible in the center of the image. The number of areas with coating loss was low, with an exposed substrate surface area of approximately 5% in relation to the total surface area of the implant. In some areas on the HA coated Ti samples, contrast differences were observed with bands of darker crystals and these areas also showed charging effects. This could be due to small amounts of organic residues which remained on the crystals and substrate after the DMSO washing. For the ZrP coated samples (Ti and PEEK), no coating loss was observed.

#### Interferometry

The interferometry results of all samples used in the different studies are shown in Table 2. Grit blasting of the Ti implants had a strong effect on the surface topography, increasing the arithmetic mean height (Sa) from 0.23 µm to 0.75 µm. The blasting also increased the developed interfacial area ratio (Sdr), from 3.71 to 15.3%. The effect on topography of the HA and ZrP coatings on top of the blasted and machined Ti samples was very low, and non-significant. The PEEK machined implants were significantly rougher than the machined Ti implants (Sa = 0.53 vs. 0.23 µm) and had a much higher interfacial area ratio (Sdr = 19.0 vs. 3.71). Similar to the effect on the Ti substrates, there were no significant differences between the HA and ZrP treated PEEK surfaces and the unmodified PEEK.

**Table 2:** Results of the interferometry analysis of the implants

Implant	Sa, µm (Sd)	Sdr, % (Sd)	Sds, 1/µm^2^ (Sd)	Used in test number
Ti, blasted	0.752 (0.14)	15.3 (0.88)	0.247 (0.08)	1
Ti, blasted + HA	0.748 (0.12)	15.0 (0.85)	0.252 (0.08)	1
Ti, blasted + ZrP	0.739 (0.21)	14.5 (0.63)	0.270 (0.06)	1
Ti, machined	0.234 (0.04)	3.71 (0.28)	0.150 (0.08)	2
Ti, machined + HA	0.245 (0.04)	3.66 (0.30)	0.154 (0.08)	2
Ti, machined + ZrP	0.244 (0.05	3.70 (0.28)	0.148 (0.09)	2
PEEK, machined	0.532 (0.03)	19.0 (0.63)	0.283 (0.06)	2
PEEK, machined + HA	0.542 (0.04)	18.6 (0.68)	0.290 (0.08)	2
PEEK, machined + ZrP	0.529 (0.05)	19.4 (0.65)	0.288 (0.04)	2

### Biomechanical testing

#### Test 1

**Fig. 11:**
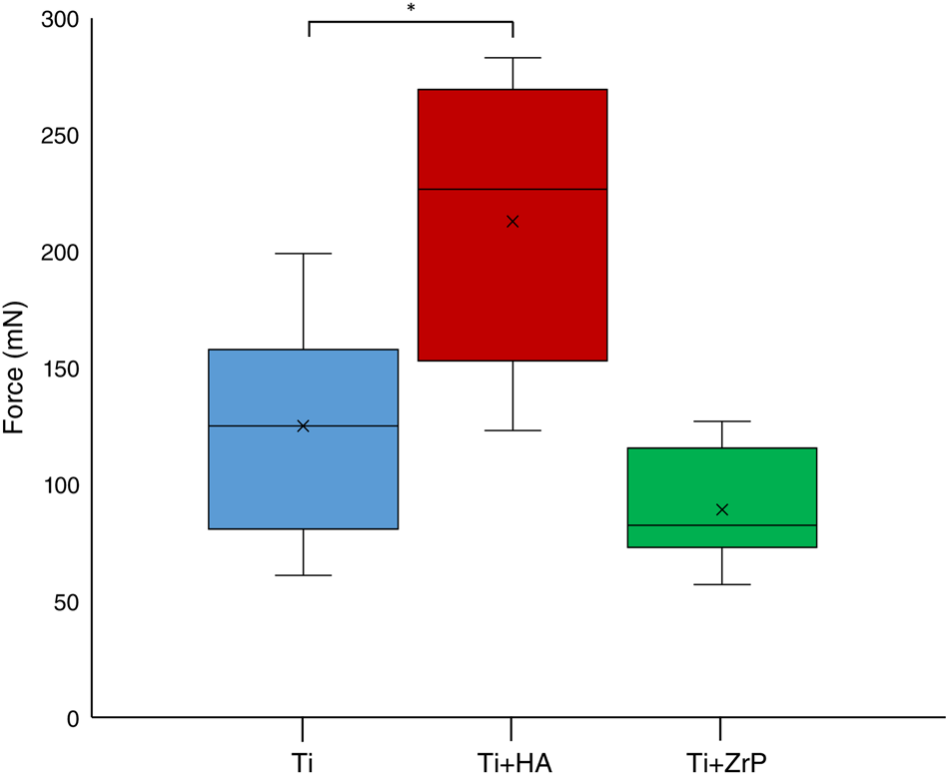
Graph (box and whisker plot) of the pullout forces from Test 1

A box and whisker plot of the pullout results from Test 1 is shown in Fig. 11. The Ti implants had a mean pullout force of 125 mN, while the HA coated Ti implants had a mean pullout force of 212 mN, (+70%, *p* < 0.01). The ZrP coated samples had a slightly lower average pullout force compared to Ti (−29%, non-significant).

#### Test 2

**Fig. 12:**
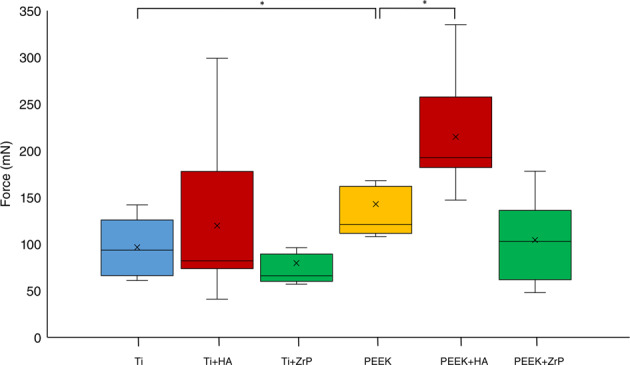
Graph (box and whisker plot) of the pullout forces from Test 2

The pullout values for Test 2 are shown in Fig. 12. The Ti+HA implants had an average higher pullout force compared to the uncoated Ti (+ 24%, non-significant). The average force for the Ti+ZrP implant group was lower than uncoated Ti (−18%, non-significant). The PEEK + HA group was higher than the uncoated PEEK (+ 51%, *p* < 0.05), while PEEK + ZrP was lower than the uncoated PEEK (−27%, non-significant). The untreated PEEK had a higher pullout force compared to Ti (+ 48%, *p* < 0.05).

### Test 2-Histomorphometry

**Fig. 13:**
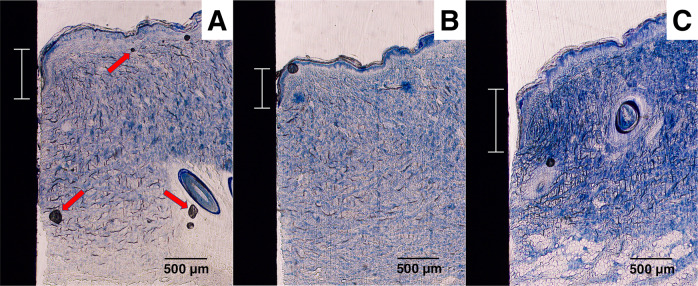
Examples of histology slides for the Ti surfaces: **A** Unmodified Ti, **B** Ti+HA, **C** Ti+ZrP. The Epidermal Downgrowth (shown with scale bar) for these samples were **A** 0.59, **B** 0.48, **C** 0.77 mm. Arrows in **A** show artefacts created by the histology processing

**Fig. 14:**
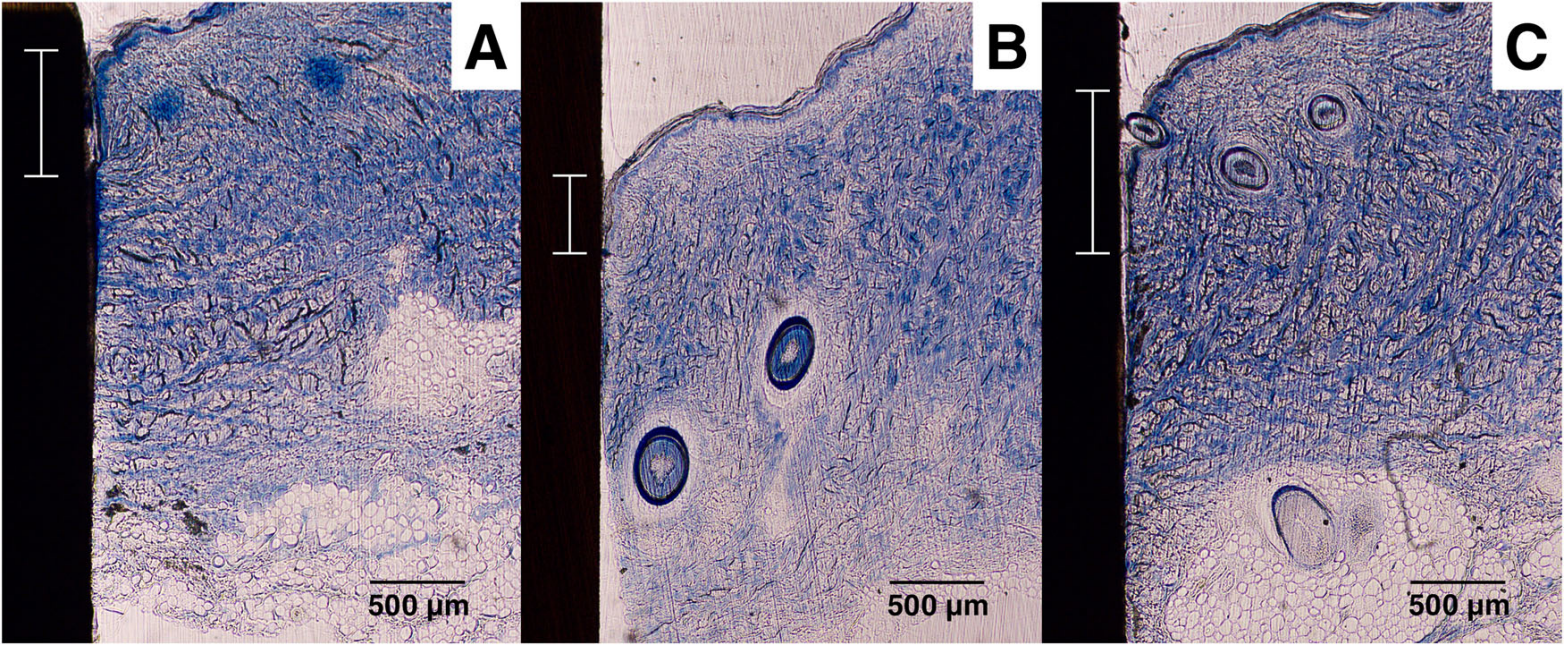
Examples of histology slides of the PEEK surfaces: **A** Unmodified PEEK, **B** PEEK + HA, **C** PEEK + ZrP. The Epidermal downgrowth (shown with scale bar) for these samples were **A** 0.68, **B** 0.43, **C** 0.89 mm

**Fig. 15:**
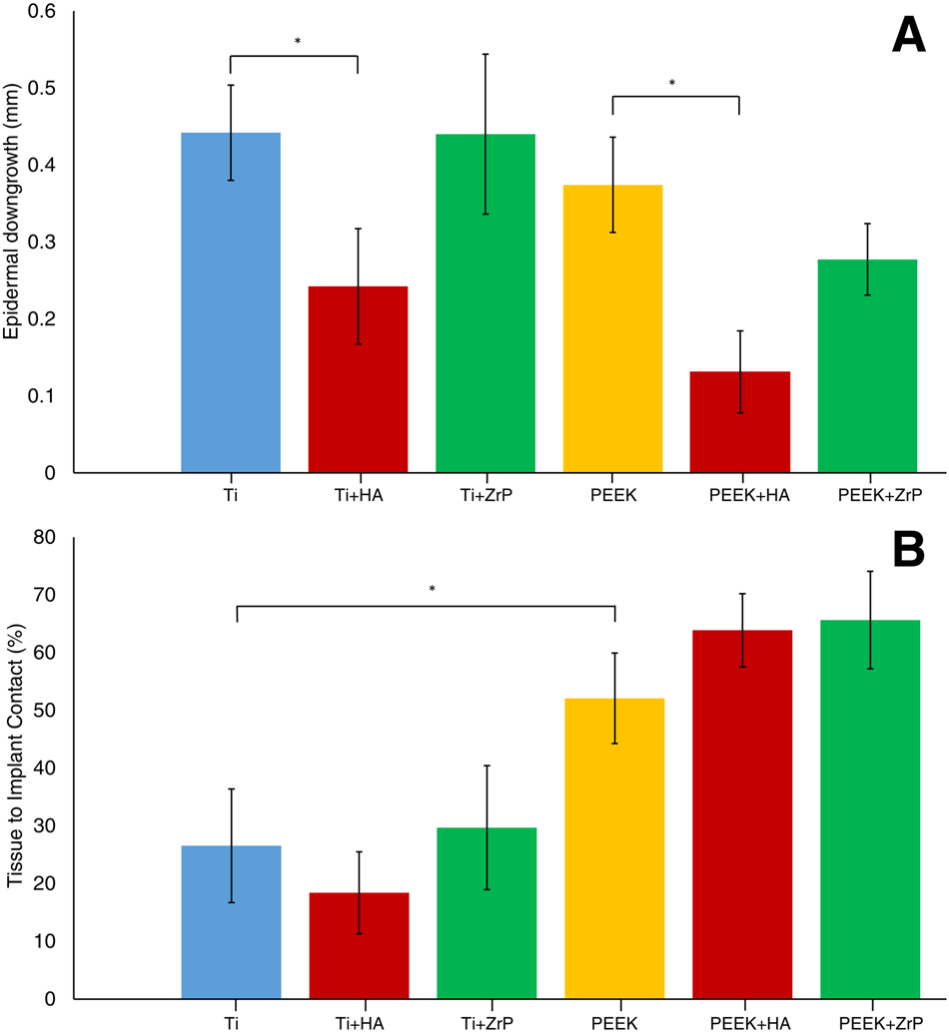
Results from the histomorphometry on the Ti and PEEK samples, **A** Epidermal Downgrowth (ED), **B** Tissue to Implant Contact (TIC). Error bars show Standard Error

The samples for histomorphometry were prepared as described in Section 2.6. Figure 13A–C show examples of histological slides of the uncoated, HA coated and ZrP coated Ti samples. In these examples, Fig. 13A (untreated Ti), Fig. 13B (Ti + HA) and Fig. 13C (Ti + ZrP) have ED values of 0.59, 0.48 and 0.77 mm, respectively. Note that these images also display artefacts (black spots), arising from the staining procedure, indicated with red arrows in Fig. 13A. Figure 14A–C show examples of histological slides of uncoated, HA coated and ZrP coated PEEK samples. In these examples, Fig. 14A (untreated PEEK), Fig. 14B (PEEK + HA) and Fig. 14C (PEEK + ZrP) have ED lengths of 0.68, 0.43 and 0.89 mm, respectively. A summary of the ED and TIC measurements is shown in Fig. 15. As seen in Fig. 15A, HA decreased the ED compared to untreated Ti (−45%, *p* < 0.05). No difference in ED compared to untreated Ti was observed for the ZrP coated implants (0%, non-significant). ED for PEEK + HA compared to untreated PEEK was decreased (−65%, *p* < 0.05). For the ZrP coated vs. uncoated PEEK the ED also decreased (−26%, non-significant). The ED for PEEK vs. Ti was slightly lower (−21%, non-significant). A summary of the TIC results is shown in Fig. 15B. For TIC, no clear pattern was observed within the Ti or PEEK groups. In the Ti implant groups the ZrP-coated Ti had the highest TIC, but the differences between the groups were not significant. In the PEEK implant group, PEEK + ZrP showed the highest TIC, but the differences were not significant. The untreated PEEK had significantly higher TIC compared to Ti (+96%, *p* < 0.05).

## Discussion

Perhaps the most important topic to be investigated in this study was the correlation between the biomechanical testing and the histomorphometry. While histomorphometry is a well-accepted method for evaluation of soft tissue integration, biomechanical testing is much less studied. For the biomechanical technique to be a valuable tool, there should be a correlation between the biomechanical testing and the histomorphometry. Table 3 shows a summary of the results. A point of concern was that an increased ED would cause an increased pullout force; the downgrowth of the epidermis would create a pressure on the implant which could increase the mechanical force required to pull out the implant. An implant with poor integration would then give a high pullout force. The histology analysis showed, however, that this was not the case. The sample groups which displayed a mean high pullout force in the biomechanical test generally showed a low epidermal downgrowth (one exception is the comparison between untreated PEEK vs. PEEK + ZrP; higher pullout force (Fig. 12) and higher ED (Fig. 15A). With regards to TIC, in theory a higher tissue to implant contact should create a higher removal force, and this parameter could be of equal importance as the ED to complement the biomechanical results. In the present study, this parameter varied too much within the individual groups to show any significance. However, there was a significant (*p* < 0.05) difference for untreated machined PEEK vs. Ti, which correlates with the mean higher pullout value for PEEK vs. Ti. One difficulty with the TIC measurement was detachment of the tissue from the implant during the histological processing. Since the adhesive forces between implant and tissue are very small, the tissue can detach from the implant, especially during the early stages of fixation and embedding. This effect is visible in Fig. 6. Judging from the very smooth appearance of the tissue below the “TC” scale bar in this Figure, (approx. 250 µm long), this tissue section was probably attached to the implant before the histological processing, and detached during the process. This source of error may explain why the TIC parameter did not show a good correlation with the pullout force.

**Table 3:** Summary of test results

Test #	Samples	Biomechanical test results	ED	TIC	SEM
1	Ti Ti + HA Ti + ZrP	Ti+HA vs. Ti: +70% ** Ti+ZrP vs. Ti: −29%	–	–	Coating loss of approx. 5% for the HA surfaces, no loss observed for the ZrP surfaces.
**2**	Ti Ti + HA Ti + ZrP P P + HA P + ZrP	Ti+HA vs. Ti:+24% Ti+ZrP vs. Ti: −18% P + HA vs. P: + 51% * P + ZrP vs. P: −27% P vs. Ti: +48% *	Ti+HA vs Ti: −45% * Ti+ZrP vs Ti: 0% P + HA vs. P: −65% * P + ZrP vs. P: −26% P vs. Ti: −21%	Ti+HA vs Ti: −31% Ti+ZrP vs Ti:+12% P + HA vs. P: + 12% P + ZrP vs. P: + 3% P vs. Ti: +96% *	Coating loss of approx. 5% for the HA surfaces, no loss observed for the ZrP surfaces.

*P* PEEK. *p < 0.05, **p < 0.01

Other parameters described in the literature to assess soft tissue integration of implants are the distance between the top of the implant to the gingiva [39] or the pocket depth between the skin and implant [24]. However, these parameters require the tissue to be smooth, and porcine skin is too irregular to make the evaluation of these parameters meaningful.

The effect of HA was similar to as reported in previous studies [10, 13–15]. These studies have used micrometer-thick coatings, whereas in the present study an ultrathin (20–40 nm thick) coating was evaluated – and it appears that this coating works as well as HA coatings which are several orders of magnitude thicker. HA also seemed to work better on rough substrates compared to smooth ones – the HA coating had significantly higher pullout value differences for coated vs. uncoated on blasted Ti (Sa = 0.75 µm) and PEEK (Sa = 0.53 µm), compared to machined Ti (Sa = 0.23 µm) where the Ti+HA average value was higher, but non-significant.

Another important result in these tests was the differences in pullout force and ED between PEEK and Ti. While the pullout tests and the histomorphometry clearly indicated that PEEK showed a better integration to the skin, the PEEK implants were rougher than the Ti implants. This may partly explain the higher measured values for PEEK, but the rough Ti that was used in Test 1 had similar roughness parameters as PEEK, and had an average pullout force of 125 mN, compared with 142 mN for the PEEK implant group in Test 2. This indicates that the integration of PEEK in soft tissue seems to be more favorable compared to the integration in bone, where Ti integrates better than PEEK [31–33, 39].

The SEM analysis showed that the ex vivo implantation for 2 weeks had no major effect on the coatings. For the HA layer, the analysis showed a loss of coating in some areas, but these areas were few, approximately 5% of the total coated surface. The pH in soft tissue will not be as low as created by the osteoclasts in bone, so it is reasonable to believe that HA placed in skin should be more resistant to degradation compared to in bone, but there are other mechanisms which may degrade HA; inflammatory response and abiotic dissolution are some examples. The ZrP surface showed no loss of coating. In bone, the coatings will behave differently, an important difference between the coatings is that whereas HA is readily dissolved by the acidic environment caused by the osteoclasts in bone, ZrP has a very low dissolution rate at mildly acidic (pH 3–6) conditions [40].

## Conclusion

A method to evaluate implant integration in explanted skin by biomechanical measurement was developed and evaluated. The results from the biomechanical tests were compared with histomorphometry. PEEK implants showed a better soft tissue integration compared to titanium (Ti), with higher pullout values, lower epidermal downgrowth and higher tissue to implant contact. This is contrary to bone tissue integration where Ti shows a better implant integration compared to PEEK. The effect of a nanosized HA coating on both PEEK and Ti was observed to have a positive effect on soft tissue integration. Thus, the beneficial effect of nanosized HA seems to be valid both for soft and hard tissue integration. Nanosized ZrP on the other hand has been shown to have a strong effect on bone tissue growth, but did not display a positive effect in this model. Overall, the biomechanical results had a good correlation with the epidermal downgrowth of the different groups; a low epidermal downgrowth resulted in a high pullout force. Thus, biomechanical testing on ex vivo cultivated porcine skin appears to be a promising approach to evaluate soft tissue integration.
